# Optimized OPA1 Isoforms 1 and 7 Provide Therapeutic Benefit in Models of Mitochondrial Dysfunction

**DOI:** 10.3389/fnins.2020.571479

**Published:** 2020-11-26

**Authors:** Daniel M. Maloney, Naomi Chadderton, Sophia Millington-Ward, Arpad Palfi, Ciara Shortall, James J. O’Byrne, Lorraine Cassidy, David Keegan, Peter Humphries, Paul Kenna, Gwyneth Jane Farrar

**Affiliations:** ^1^The School of Genetics & Microbiology, Trinity College Dublin, Dublin, Ireland; ^2^National Centre for Inherited Metabolic Disorders, The Mater Misericordiae University Hospital, Dublin, Ireland; ^3^Clinical Genetics Centre for Ophthalmology, The Mater Misericordiae University Hospital, Dublin, Ireland; ^4^The Research Foundation, Royal Victoria Eye and Ear Hospital, Dublin, Ireland

**Keywords:** optic atrophy 1, dominant optic atrophy, mitochondria, gene therapy, optic neuropathy, AAV, bioenergetics, retinal ganglion cells

## Abstract

Optic Atrophy 1 (OPA1) is a mitochondrially targeted GTPase that plays a pivotal role in mitochondrial health, with mutations causing severe mitochondrial dysfunction and typically associated with Dominant Optic Atrophy (DOA), a progressive blinding disease involving retinal ganglion cell loss and optic nerve damage. In the current study, we investigate the use of codon-optimized versions of OPA1 isoform 1 and 7 as potential therapeutic interventions in a range of *in vitro* and *in vivo* models of mitochondrial dysfunction. We demonstrate that both isoforms perform equally well in ameliorating mitochondrial dysfunction in OPA1 knockout mouse embryonic fibroblast cells but that OPA1 expression levels require tight regulation for optimal benefit. Of note, we demonstrate for the first time that both OPA1 isoform 1 and 7 can be used independently to protect spatial visual function in a murine model of retinal ganglion cell degeneration caused by mitochondrial dysfunction, as well as providing benefit to mitochondrial bioenergetics in DOA patient derived fibroblast cells. These results highlight the potential value of OPA1-based gene therapy interventions.

## Introduction

Optic Atrophy 1 (OPA1) is a mitochondrially targeted GTPase that plays a pivotal role in mitochondrial health, with mutations causing severe mitochondrial dysfunction. The OPA1 protein possesses a mitochondrial targeting sequence and localizes to the inner mitochondrial membrane (IMM), where it promotes fusion of the IMM by interaction with cardiolipin on the opposing section of IMM (Ban et al., 2017; Lee and Yoon, 2018). OPA1 is sufficient for IMM fusion but in order to successfully form tubular mitochondrial networks OPA1 works in conjunction with two other GTPases, Mitofusin 1 and 2 (*MFN1* and *MFN2*), which mediate outer mitochondrial membrane fusion (Song et al., 2009). The process of mitochondrial fusion is counter-balanced by mitochondrial fission mediated by a fourth GTPase, dynamin 1-like protein (*DNM1L*) (Smirnova et al., 1998; Macdonald et al., 2016). Independent of its role in mitochondrial fusion, OPA1 has also been shown to be anti-apoptotic as OPA1 disassembly mediates the release of cytochrome c from the mitochondria (Yamaguchi et al., 2008), potentially due to the role OPA1 plays in regulating cristae junctions (Frezza et al., 2006). Furthermore OPA1 protein is needed for the correct maintenance of the mitochondrial genome (Chen et al., 2010), as well as playing a role in the organization of the mitochondrial supercomplexes (Cogliati et al., 2013; Lee et al., 2017). Recently OPA1 has been implicated in regulating DNA methylation in human neuronal development (Caglayan et al., 2020) and also in the spatial arrangement of the mitochondria in adjacent photoreceptor cells (Meschede et al., 2020).

The *OPA1* gene consists of 31 exons, which produce 8 mRNA isoforms that differ due to the alternate splicing of exons 4, 4b, and 5b (Delettre et al., 2001). Once located to the mitochondrial IMM, full length OPA1 protein (l-form) can be proteolytically cleaved by either OMA1 or YME1L at sites on exon 5 or 5b, respectively (Song et al., 2007; Ehses et al., 2009). These l and s-forms have been shown to play distinct physiological roles. Expression of any of the eight isoforms was sufficient to restore mtDNA levels, reorganize mitochondrial cristae, and boost the function of the electron transport chain in OPA1 knock out cells. However, the presence of both l and s-forms of OPA1 are needed to fully restore wild-type mitochondrial physiology (Del Dotto et al., 2017). OPA1 mRNA isoforms show a tissue specific expression pattern but to date human retinal tissue has not been directly examined for OPA1 isoform expression (Olichon et al., 2007; Akepati et al., 2008).

Dominant Optic Atrophy (DOA) is an inherited blinding disease that primarily targets the retinal ganglion cells (RGC). DOA has an estimated prevalence of between 1–10,000 and 1–30,000, making it one of the most common optic neuropathies (Yu-Wai-Man et al., 2010; Lenaers et al., 2012). DOA typically manifests in the first or second decade of life, with progressive bilateral visual loss with central scotomas, decreased thickness of the retinal nerve fiber layer (RNFL) and optic nerve damage (Chun and Rizzo, 2017). Around 20% of DOA patients show a multi-system disorder characterized by hearing loss, ataxia, myopathy, late onset cardio myopathy and peripheral neuropathy called DOA plus (Skidd et al., 2013). Mutations in *OPA1* are commonly associated with DOA, with around 65–90% of cases due to mutations in the *OPA1* gene and around 370 variants in *OPA1* associated with the disease (Delettre et al., 2000; Del Dotto et al., 2018a). DOA patient derived fibroblasts have previously been shown to suffer significant levels of mitochondrial dysfunction showing decreased mitochondrial oxygen consumption rates, decreased ATP levels, fragmented mitochondria, mtDNA depletion and dysfunctional mitophagy, but the exact phenotypic manifestation depends on the particular mutation (Belenguer and Pellegrini, 2013; Vidoni et al., 2013; Liao et al., 2017).

Significant research has been undertaken highlighting the potential use of OPA1 as a therapeutic entity, both as a therapeutic for DOA and other mitochondrial associated diseases, as well as other more general apoptotic insults. A mouse model constitutively expressing OPA1 showed increased mitochondrial supercomplex formation, as well as protection from reperfusion ischemia damage and mitigation of the deleterious effects of the *Ndufs4*^−^*^/^*^–^ and *Cox15* mouse models (Cogliati et al., 2013; Civiletto et al., 2015; Varanita et al., 2015). The use of l-form OPA1 has been shown to alleviate acute ischemic stroke injury in rat brain, preventing neuronal cell loss (Lai et al., 2020).

AAV delivered OPA1 isoform 1 showed significant protection of RGCs in a mouse model heterozygous for a pathogenic *Opa1* mutation (Opa1 delTTAG/ +), but did not show a significant increase in visual acuity (Sarzi et al., 2018). In addition, it is notable that AAV delivered OPA1 has also been shown to be beneficial in a laser induced model of glaucoma in rats, where it provided protection of RGCs (Hu et al., 2018) and in a chemical model of ocular mitochondrial uncoupling (Sun et al., 2016b).

In this study we have explored the potential of two codon optimized *OPA1* isoforms, 1 and 7, to rescue mitochondrial dysfunction in a range of *in vitro* and *in vivo* models, including DOA patient derived fibroblasts and a rotenone induced mouse model of ocular complex 1 deficiency (Zhang et al., 2002). Rotenone irreversibly inhibits Complex 1 leading to significant RGC loss, RNFL thinning and a progressive decrease in visual acuity, thus phenotypically resembling optic neuropathies such as DOA, and Leber Hereditary Optic Neuropathy. Notably, AAV-mediated intravitreal delivery resulted in significant protection of visual function in rotenone treated animals. Furthermore, DOA patient derived fibroblasts treated with the same AAVs showed a significant improvement in mitochondrial bioenergetics.

## Materials and Methods

### RNAseq Data Analysis

RNAseq data of healthy human retina was uploaded by (Li et al., 2014) and accessed through the NCBI Gene Expression Omnibus (GEO; Accession: GSE94437, mean age = 74). All 16 retinal samples were used, 8 macular retinal samples and 8 peripheral retinal samples. 26 healthy human Dorsolateral prefrontal cortex samples were acquired from GSE80655 (Ramaker et al., 2017, mean age = 48). 19 healthy human skeletal muscle samples were acquired from GSE129643 (Ubaida-Mohien et al., 2019, mean age = 56). All samples were from Caucasian donors.

Sequence data were analyzed using Kallisto Quant (Bray et al., 2016) on the Galaxy platform (usegalaxy.org, Afgan et al., 2018) to generate RNA isoform abundance tables.

### Expression Construct Synthesis and Cloning

The OPA1 isoform 1 and isoform 7 cDNA sequences were obtained from NCBI (OPA1 isoform 1 Accession: CCDS43186.1; OPA1 isoform 7 Accession: CCDS33917). The coding sequences were both codon optimized (GeneArt, Thermo Fisher Scientific, United States) and a 6x HIS tag appended before the stop codon. A minimal rabbit β-globin poly(A) signal (Levitt et al., 1989) and flanking restriction enzyme sites, to aid subsequent cloning, were also added. The resulting sequences were synthesized by IDT (United States) and cloned into the pCMV-MCS expression plasmid (GenBank Accession: AF369966.1) using appropriate restriction enzymes (New England Biolabs, United States) as per the manufacturers protocol.

### Cell Culture

OPA1^–/–^ mouse embryonic fibroblast (MEF) cells, developed by the Chan lab (Chen et al., 2003; ATCC, CRL-2995) and NIH-3T3 cells (ECACC; 9301524) were routinely cultured in either glucose medium [DMEM GlutaMax (Thermo Fisher Scientific, United States; 61965-026) which contains 25 mM Glucose supplemented with 10% FBS (Merck; F7524) and 1 mM Sodium Pyruvate (Thermo Fisher Scientific, United States; 11360.039)] or Galactose medium (DMEM (Thermo Fisher Scientific, United States; A1443001) supplemented with 10 mM Galactose (Merck; G5388), 10% FBS, 1 mM Sodium Pyruvate and 2 mM L-Glutamine (Thermo Fisher Scientific, United States; 25030081).

### Primary Cell Lines

Patient derived primary fibroblasts were isolated from biopsies obtained with prior informed consent from patients with genetically confirmed and clinically diagnosed DOA associated visual dysfunction. DOA 1 (46 y/o, Female) has a heterozygous 53del10 deletion and DOA 2 (49 y/o, Female) and DOA 3 (18 y/o, Female) have heterozygous 1334G > A mutations. DOA 3 has primarily an eye phenotype at 18 years while DOA 2 has developed a more severe phenotype at age 49 years including optic atrophy, bilateral ptosis, progressive external ophthalmoplegia, ataxia and peripheral sensory neuropathy without cardiac involvement.

Control fibroblast cell lines were from individuals with no history of visual or mitochondrial dysfunction. Control 1 was derived from a female aged 32, control 2 was derived from a male aged 35 and control 3 was from a female aged 40.

### Plasmid Transient Transfection

Cells were transiently transfected using Lipofectamine 2000 (Thermo Fisher Scientific, United States; 11668019) as per the manufacturer’s protocol, with adjustments to account for differences in plasmid size to ensure equal plasmid copy numbers between groups. All cells were transfected 24 h after seeding when cells were at ∼70% confluency. Assays were performed 48 h post transfection.

### Cell Culture Histology

After appropriate treatments, culture medium was removed and cells were washed with PBS. Cells were fixed with 4% paraformaldehyde (pfa) for 20 min at room temperature. Cells were washed three times with PBS, before being blocked (5% Donkey Serum and 0.3% triton in PBS) at room temperature (RT) for 2 h. Primary antibody staining was carried out in blocking solution using Anti-6xHIS (1:500; Abcam; ab9108), at 4°C overnight. Secondary staining was carried out in blocking solution using Alexa Fluor 488 Anti-Rabbit (1:400; Jackson ImmunoResearch; 711-545-152) for 2 h at RT. Nuclei were stained with DAPI 1:50,000 in PBS for 10 min at RT.

### Stable Cell Line Generation

The OPA1 isoform 1 and 7 cDNAs were cloned into pcDNA3.1 (+) (Thermo Fisher) and transfected into OPA1^–/–^ MEF cells as above. Successfully transfected cells were then selected for with 200 μg/ml G418 (Santa-Cruz Biotechnology, Inc., United States). After 2 weeks of selection cells were serially diluted and seeded at ∼1 cell per well in a 96 well plate, in the presence of G418. Wells containing a single cell were identified and split into 24 then 6 well plates before being assessed for OPA1 isoform 1 and 7 construct RNA and protein expression. Positive cell lines were then maintained under routine cell culture conditions, with the addition of G418. As a control, a stable OPA1^–/–^ MEF cell line was also generated using an empty pcDNA3.1 (+) plasmid.

### Mitotracker Staining of Mitochondria and Morphological Analysis

Mitotracker CMTM Orange (M7510, Thermo Fisher Scientific) was used as per the manufacturers protocol. Briefly, a stock solution of Mitotracker was reconstituted to 1 mM with DMSO. The stock solution was then diluted to a working concentration of 100–500 nM in pre-warmed complete medium. Medium was removed from cells and 1 ml of Mitotracker staining solution added. Cells were incubated at 37°C with 5% CO_2_ for 30 min. Cells were then washed once in complete media or were fixed and stained as previously described.

For analysis of mitochondrial morphology 10 random fields of view (FOV) were taken of three separate wells for each cell type after Mitotracker staining using an Olympus IX83 inverted motorized microscope. Images were analyzed blind and scored by eye for any cells that demonstrated any tubular branching mitochondria and were reported as a proportion of all cells per FOV.

### Cell Growth Analysis

Cells were seeded in a 6 well plate at 1 × 10^6^ cells per well with either glucose or galactose medium. 48 h later cells were trypsinised and cell number was determined using trypan-blue exclusion on a Luna Cell Counter (Logos Biosystems). Trypsinised cells were then reseeded at 1 × 10^6^ and the process was repeated for a total of 192 h.

### Seahorse Mitochondrial Metabolism Assays

The Seahorse XFe96 extracellular flux analyser (Agilent) was used to assay a number of mitochondrial metabolic parameters. For the Mitochondrial Stress Test of OPA1^–/–^ MEFs and stable cell lines, cells were seeded 24 h prior to the assay in Seahorse Biosciences 96 well plates at 2.5 × 10^4^ cells per well in 80 μl of complete glucose or galactose DMEM. One hour prior to the assay, culture media was substituted for Seahorse XF DMEM medium, pH7.4 (Agilent; 103575-100), supplemented with 1 mM Sodium Pyruvate, 2 mM L-Glutamine and either 25 mM Glucose or 10 mM Galactose, and placed in a non-CO_2_ 37°C incubator. The Mitochondrial Stress Test was performed by the sequential addition of 1 μM Oligomycin, 1 μM FCCP, and 0.5 μM Rotenone and Antimycin A.

The above protocol was modified for use with primary fibroblasts. Cells were seeded 72 h prior to the assay at 5 × 10^3^ cells per well in complete glucose or galactose DMEM. 24 h later media was replaced with 50 μl 2% FBS DMEM GlutaMAX (containing glucose or galactose and 1 mM Sodium Pyruvate) as cells were transduced with AAV at various multiplicities of infection (MOIs). After a further 12 h an equal volume of 18% FBS DMEM was added. The Mitochondrial Stress Test was performed 48 h after AAV transduction as described above, but with 2.25 μM FCCP instead.

The ATP Rate Assay was carried out as per the manufacturers protocol (Agilent; 103592-100) using 2.5 × 10^4^ OPA1^–/–^ MEF or stable cells per well. Briefly, cells were sequentially treated with 1.5 μM Oligomycin then 0.5 μM Rotenone and Antimycin A. Rates were calculated using the ATP Rate Assay plug-in for the Wave software package.

Data from Seahorse runs were normalized either to cell count or using the Bradford method of protein quantification as per the manufacturers protocol (Thermo Fisher Scientific; Cat. No. 23246)

### Mitochondrial Fusion Assay

Mitochondrial fusion assay was adapted from Karbowski et al. (2014). Briefly, 5 × 10^5^ cells were seeded into a 35 mm μ-Dish (iBidi; 81156) 72 h prior to the assay. 24 h later cells were transfected with a mito-PAGFP plasmid (Richard Youle; Addgene plasmid #23348; Karbowski et al., 2004) and a mito-dsRED plasmid (Michael Davidson; Addgene plasmid # 55838). The presence of mito-dsRED allows transfected cells to be identified. The analysis was performed on a Leica SP8 gated STED confocal microscope where cell dishes were placed in the incubated stage and given 10 min to equilibrate to temperature. Five mito-dsRED positive FOV were then identified, and their stage co-ordinates marked. Z-stack limits were then set for each of the target cells. The five FOV are imaged as T = −1. A 2.5 μm × 2.5 μm region of interest (ROI) was then demarcated on an area rich in mitochondria in each cell. The microscope was then set to illuminate only the ROI (background set to 0%) with the UV lamp, with lamp power set to 40%. Sequentially, all cells were then illuminated with the UV lamp in the predefined ROI. Cells were imaged immediately as T0. Cells were imaged further every 15 min for a total of 45 min.

Analysis of the images was completed using Leica Application Suite X. Two 2.5 μm × 2.5 μm ROIs were taken for each cell at each timepoint, one measuring the fluorescent intensity of the area that received UV illumination, and a second ROI elsewhere in the cell that did not receive any illumination. The intensity levels for the green channel (PA-GFP) were recorded. The green value in the unilluminated ROI for a given cell was used to normalize the intensity levels of the cell to other cells, accounting for fluctuations in protein expression between cells. The overall level of fission and fusion was expressed as the percentage of green signal that remained in the UV illuminated ROI after 45 min (T = 45), when compared to T = 1.

### Peredox Assay for NADH Level

The change in NADH level in cells was estimated using the Peredox fluorescent protein as outlined in Hung et al. (2011). Briefly, cells were seeded in 8 well chamber slides suitable for confocal imaging. Twenty four hours later cells were transfected with the pcDNA3.1-Peredox-mCherry plasmid [Gary Yellen (Addgene plasmid # 32383)]. 48 h later cells were imaged with a Carl Zeiss LSM 710 confocal microscope using the heated stage at 37°C and 5% CO_2_. Random FOV were selected and cells were imaged using the red and green channels using consistent imaging settings. Image analysis was carried out using ZEN blue edition software by Carl Zeiss AG. An ROI was drawn in each cell and the intensity of green and red signals recorded. The green signal, corresponding to NADH level, was then normalized to the red signal, corresponding to Peredox protein level.

### Western Blotting

Protein was extracted from cells after washing with ice-cold PBS using RIPA buffer supplemented with cOmplete mini Protease inhibitor cocktail (Roche; 11836153001). Crude lysates were incubated on ice for 30 min with occasional vortexing before centrifugation at 14,000G at 4°C for 30 min. Supernatants were then aliquoted for protein quantification via the Bradford method and stored at −80°C.

Extracted protein was separated by 4–12% SDS-PAGE gel electrophoresis and semi-dry transferred to PVDF membranes (Merck; IPVH00010). Membranes were blocked in 5% non-fat milk at room temperature for 1 h before overnight incubation with primary antibody in blocking solution [Anti-6xHIS (1:1,000; Abcam; ab9108), OPA1 (1:500; Abcam; ab90857) or β-actin (1:5,000; Abcam; ab8227)]. Blots were incubated with Anti-Rabbit Peroxidase secondary antibody (1:10,000; Sigma; A9169) followed by exposure using an enhanced chemiluminescence (ECL) kit (Thermo Fisher Scientific; 32209 or Advansta; K-12045-D20) before imaging with the C-DiGit Blot Scanner (Li-Cor) or being developed in a darkroom. Densitometry analysis was carried out using the Fiji distribution of Image J (Schindelin et al., 2012).

### Real-Time RT-PCR

RNA was extracted from cells using an RNeasy kit (Qiagen; 74104). OPA1 and codon optimized OPA1 (huOPA1) mRNA expression levels were assayed via real-time RT-PCR using a StepOnePlus system (Applied Biosystems) with QuantiTect SYBR Green RT-PCR kit (Qiagen; 204245). Cells from 4 virally treated wells were pooled and all samples were run in triplicate. Primers were designed to target all 8 endogenous OPA1 mRNA isoforms (All isoforms OPA1: F 5′-AGTAGAGGTTGCTTGG GAGAC-3′ and R 5′-TGTCATCATGCTCTTTCCCT-3′) and a separate pair were designed to target both optimized OPA1 iso 1 and 7 (optOPA1: F 5′- TACCCCAGACTGAGAGAGCT-3′ and R 5′- ACTTGGCTCAGGGAGATCAC-3′). β-actin levels were used as an endogenous control (β-actin: F 5′- TCA CCCACACTGTGCCCATCTACGA-3′ and R 5′-CAGCG GAACCGCTCATTGCCAATGG-3′). To obtain copy numbers of both endogenous OPA1 and optimized OPA1 transgene, a standard curve was generated from a plasmid of known copy number containing fragments of both wild type OPA1 and codon optimized OPA1 versions.

### AAV production

AAV2/2-OPA1 iso 1, AAV2/2-OPA1 iso 7 and AAV2/2-CAG EGFP were prepared by the Farrar group at TCD (Lane et al., 2020). The two OPA1 cDNAs were cloned into pAAV-MCS (accession no. AF396260.1; Agilent Technologies, Inc., United States).

Recombinant AAV2/2 viruses were generated by helper virus free, triple transfection based on the method described by (Xiao et al., 1998). Human embryonic kidney cells (accession number CRL-1573; ATCC, United States) were transfected with pAAV-MCS plasmids containing OPA1 iso 1 or 7, pRep2/Cap2 and pHelper (Agilent Technologies, Inc., United States) at a ratio of 1:1:2, using polyethylenimine, as previously described (O’Reilly et al., 2007). 72 h post-transfection, AAV particles were purified from the clarified lysate by cesium gradient centrifugation. AAV containing fractions were dialyzed against PBS supplemented with Pluronic F68 (0.001%; Bennicelli et al., 2008). Genomic titers (viral genomes/ml; vg/ml) were determined by quantitative real-time PCR (Rohr et al., 2002).

### Intravitreal Injections

All animal work was performed in accordance with the European Union (Protection of Animals used for Scientific Purposes) Regulations 2012 (S.I. no. 543 of 2012) and the Association for Research in Vision and Ophthalmology (ARVO) statement for the use of animals. Adult wild type 129 S2/SvHsd mice (Harlan UK Ltd., Oxfordshire, United Kingdom) were maintained in a specific pathogen free (SPF) facility. Adult mice were anaesthetized by intraperitoneal injection of medetomidine and ketamine (0.5 mg and 57 mg/kg body weight, respectively). Pupils were dilated with 1% tropicamide and 2.5% phenylephrine. Using topical anaesthesia (Amethocaine), a small puncture was made in the sclera. A 32 gauge blunt-ended microneedle attached to a 10 μl Hamilton syringe was inserted through the puncture, and 3 μl AAV2/2-OPA1-iso 1 or AAV2/2-OPA1-iso 7 (1 × 10^9^ vector genomes) plus 1 × 10^8^ AAV2/2 CAG-EGFP was slowly, over a 2-min period, administered into the vitreous of both eyes. Following intravitreal injection, an anaesthetic reversing agent (Atipamezole Hydrochloride, 1.33 mg/kg body weight) was delivered by intraperitoneal injection. Body temperature was maintained using a homeothermic heating device. 3 weeks later, 0.6 μl of 1.5 mM rotenone in DMSO was administered by the same method of intravitreal injection to one eye. Animals were sacrificed by CO_2_ asphyxiation.

The sex breakdown of each group were as follows: OPA1 iso 7 ± rotenone: 5F + 5M, Wt ± rotenone (OPA1 iso 7 cohort): 5F + 2M, Opa1 ± rotenone: 4F + 3M and Wt ± rotenone (OPA1 iso 1 cohort): 3F + 5M.

### Photopic Negative Response (PhNR)

Two weeks post rotenone the cohort of mice underwent assessment of the cone electroretinogram (ERG) Photopic Negative Response (PhNR). Detection was enhanced with an orange filter. The PhNR, the first negative deflection following the cone b wave response, was evaluated under photopic conditions using a Roland Consult RetiScan ERG RetiPort electrophysiology unit. Mice were anaesthetized as described above. PhNR responses were recorded simultaneously from both eyes by means of goldwire electrodes (Roland Consulting, Brandenburg-Wiesbaden, Germany). Standardized flashes of light were presented in a Ganzfeld bowl. Cone-isolated responses were recorded to the maximal intensity flash (−25 dB maximal intensity where maximal flash intensity was 3 candelas/m^2^/s). Following accepted convention, the initial positive deflection was termed N1, the subsequent negative deflection P1 and the next positive deflection N2. The PhNR was calculated as the negative difference between N1 and P1. The recording was only deemed successful if this was indeed negative and the trace did not deflect above N2 for the remainder of the recording.

### Optokinetic Response (OKR)

One week post PhNR assessment (3 weeks post rotenone injection), mice underwent optokinetic analysis as previously described (Chadderton et al., 2013). OKR spatial frequency thresholds were measured blind using a virtual optokinetic system (VOS, OptoMotry, Cerebral Mechanics, Lethbridge, Alberta, Canada; Prusky et al., 2004). Briefly, a virtual-reality chamber was created with four 17-inch computer monitors facing inwards and the unrestrained mouse was placed on a platform in the center. A video camera, situated above the animal, provided real-time video feedback. The experimenter centered the virtual drum on the mouse’s head and judged whether the mouse made slow tracking movements with its head and neck. OptoMotry measures the threshold of the mouse’s optokinetic tracking response to moving gratings. The visual capabilities of each eye can be measured simply by changing the direction of rotation as only rotation in the temporal-to-nasal direction evokes a tracking response (Douglas et al., 2005). The spatial frequency threshold, the point at which the mouse no longer tracked, was obtained by incrementally increasing the spatial frequency of the grating at 100% contrast. A staircase procedure was used in which the step size was halved after each reversal, and terminated when the step size became smaller than the hardware resolution (∼0.003 cyc/deg, 0.2% contrast). One staircase was presented for each direction of rotation to measure each eye separately, with the two staircases being interspersed. OKRs for each mouse were measured 3–4 times on separate days, averaged and SD values calculated.

### Retinal Ganglion Cell counts

Mice were sacrificed 3 days after OKR assessment and eyes were enucleated and fixed in 4% paraformaldehyde in PBS overnight. Eyes were washed in PBS, then the retinas were removed from the eyecups and immediately processed for immunocytochemistry. Immunocytochemistry was performed as described previously (Palfi et al., 2016). Whole retinas were incubated with primary antibodies for RBPMS (ABN1376, Millipore, 1:200; Rodriguez et al., 2014) overnight for 3 days at 4°C. Retinas were then washed in PBS and incubated with secondary antibodies conjugated with Alexa-Fluor-488, Cy3 (Jackson ImmunoResearch Laboratories; 1:400) for 2 days and nuclei counterstained with DAPI. Samples were covered using Hydromount (National Diagnostics). Fluorescent microscopy was carried out utilizing an Olympus IX83 inverted motorized microscope (cellSens v1.9 software) equipped with a SpectraX LED light source (Lumencor) and an Orca-Flash4.0 LT PLUS/sCMOS camera (Hamamatsu). Samples were imaged using a 10x plan fluorite objective utilizing enhanced focal imaging (EFI) with typically 5–8 Z-slices. Lateral frames were stitched together and analyzed in cellSens. Automated cell staining area was calculated utilizing 2D deconvolution, manual threshold and object size filter in cellSense; the same settings/operations were applied to all images.

### Data Handling and Statistics

Data handling was performed in either RStudio [RStudio Team (2015), RStudio: Integrated Development for R. RStudio, Inc., Boston, MA^1^)] or Microsoft Excel 2016. Graphs were created using either ggplot2 (Wickham, 2016) or Microsoft Excel. All statistical analysis was carried out in R. Unless otherwise stated, Kruskal-Wallis testing with pairwise Wilcoxon signed rank test *post hoc* and Bonferroni-Holm correction was used, with *p* < 0.05 considered statistically significant. Data are reported ± SD unless otherwise stated.

## Results

### Retinal OPA1 Isoforms

**FIGURE 1:**
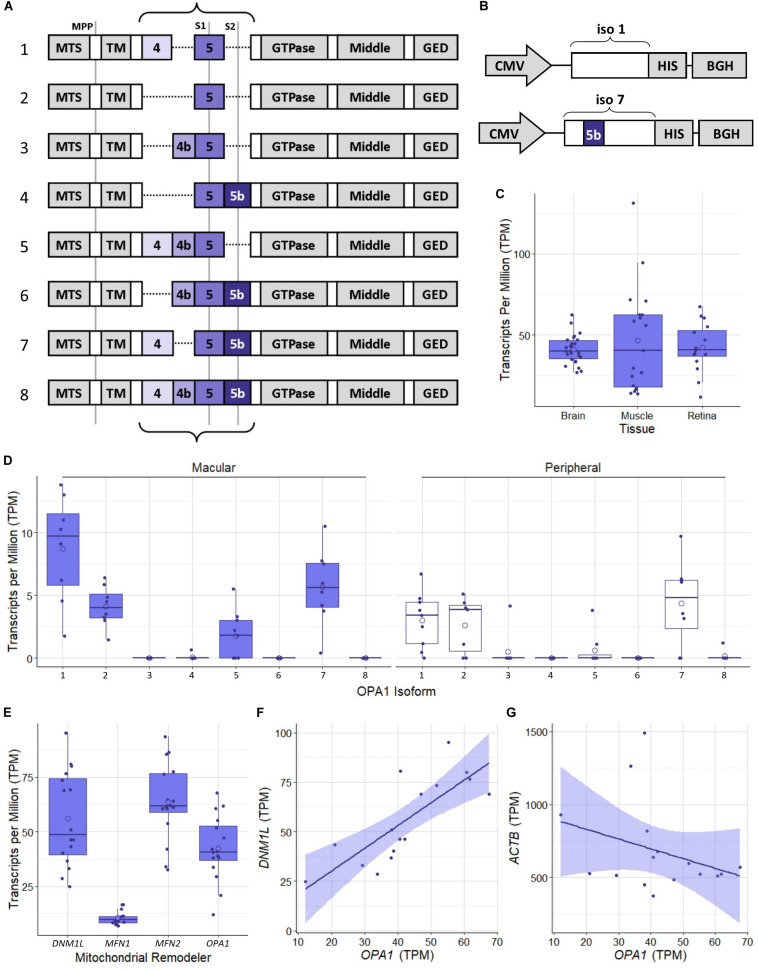
Schematic representation of OPA1 RNA isoforms and RNAseq analysis of healthy human tissue. **(A)** The 8 primary protein coding mRNA variants of OPA1 in humans. The curly braces indicate the area that undergoes alternate splicing of exons 4, 4b, and 5b. Gray lines indicate protease cleavage sites, MPP = matrix processing peptidases, S1 = OMA1 and S2 = YME1L. Gray blocks outside of the curly braces indicate functional domains; MTS, Mitochondrial Targeting Sequence; TM, Transmembrane domain; GED, GTPase effector domain. **(B)** Schematics of optimized OPA1 isoform 1 or 7 expression cassettes. CMV, Cytomegalovirus promoter; HIS, 6x HIS tag; BGH, Bovine Growth Hormone polyadenylation signal. Note that only exon 5b differs between them. **(C)** Comparison of *OPA1* transcript levels in Transcripts Per Million (TPM) in healthy dorsolateral prefrontal cortex brain tissue (*n* = 26 samples), skeletal muscle tissue (*n* = 19) and retinal tissue (*n* = 16). The open circle on each boxplot represents the mean. **(D)** Expression levels of the 8 protein coding isoforms of *OPA1* as identified in the ensemble database (ENSG00000198836) measured in TPM. *n* = 8 macular samples and *n* = 8 peripheral samples. **(E)** RNA transcript expression levels in TPM of 4 significant mitochondrial remodeling proteins Drp1 (*DNM1L*), Mitofusin 1 and 2 (*MFN1* and *2*) and *OPA1*. *n* = 16 (macular and peripheral) samples. **(F,G)** Are representative scatter plots of the correlation between mitochondrial remodeler RNA transcripts, with *ACTB* as a control. *OPA1* transcript expression is compared to either *DNM1L* or *ACTB* transcript levels, measured in TPM. *DNM1L* and *OPA1* transcript levels show a significant positive correlation (*r* = 0.79, Pearson’s Correlation Coefficient, *n* = 16, *p* < 0.05), whereas *ACTB* and *OPA1* transcript levels show no significant correlation (*r* = –0.32, Pearson’s Correlation Coefficient, *n* = 16, *p* > 0.05). Shaded region represents 95% confidence interval, BH correction for multiple testing.

The human *OPA1* gene has been identified as having a number of isoforms which have been shown to be expressed at differing levels across tissue types (Olichon et al., 2007) which are generated by alternative splicing of exons 4, 4b, and 5b (Figure 1A). Initial proteomic work has identified OPA1 isoform 1 as the primary isoform present in mouse tissue (Akepati et al., 2008). However, to the best of our knowledge the OPA1 isoform expression profile in the healthy human retinal tissue has not been investigated. In order to address this, we analyzed publicly available RNAseq data from retinal tissue from the macular retina and peripheral retina of 8 healthy post-mortem retinae (Li et al., 2014; Gene Expression Omnibus, Accession: GSE94437), as well as 26 brain tissue samples from the Dorsolateral prefrontal cortex (Ramaker et al., 2017; GSE80655) and 25 skeletal muscle samples (Ubaida-Mohien et al., 2019; GSE129643) from healthy individuals. Figure 1C shows that RNA levels of OPA1 are broadly similar across the three tissues analyzed. Figure 1D shows the transcripts per million data for the 8 protein coding isoforms of OPA1 identified in the Ensembl database (Yates et al., 2020; ENSG00000198836) in the 8 macular and 8 peripheral retinal samples. OPA1 isoforms 1 and 7 are the most highly expressed in these retinal samples, but differences between macular and peripheral retinal samples were present.

To investigate the relationship between mitochondrial remodeling proteins further, the relative expression of *DNM1L*, *MFN1, MFN2*, and *OPA1* was compared (Figure 1E), demonstrating that the RNA transcripts for *DNM1L*, *MFN2*, and *OPA1* are all expressed at similar levels in retinal cells, whereas *MFN1* levels are substantially lower than the others. To investigate any relationship between transcript expression levels, correlation coefficients were calculated between each pair of transcripts, and with *ACTB* as a control (Table 1). There is a significant positive correlation between transcript levels of *MFN2*, *DNM1L* and *OPA1* (Pearson’s correlation coefficient, Bonferroni Correction *p* < 0.01, *n* = 16), all showing around *r* ≈0.8 correlation coefficient. However, there is no significant correlation between *ACTB* transcript levels and those of the mitochondrial remodeling proteins. Figures 1F,G shows representative scatter plots demonstrating the correlation between *OPA1* and *DNM1L* transcript levels as well as *OPA1* and *ACTB* levels. All comparisons can be seen in Table 1.

**TABLE 1:** Pearson’s correlation coefficient of the RNA transcript levels of four known mitochondrial remodeler proteins with β-actin as a control from RNAseq of human retinal punches (Li et al., 2014).

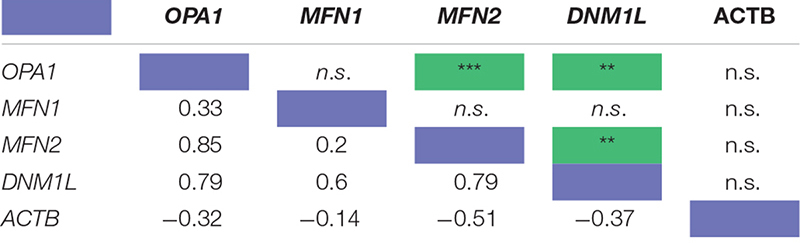

Numbers below the purple diagonal represent Pearson’s r, with significance values above the orange diagonal with Bonferroni-Holm correction. (n = 16 samples, macular and peripheral). **p < 0.01 and ***p < 0.001.

As OPA1 isoforms 1 and 7 were shown to be the most highly expressed in retinal tissue, in line with previous studies of non-ocular human tissues (Olichon et al., 2007), these two isoforms were tested for utility as a therapeutic intervention. Two expression cassettes were constructed for this purpose (Figure 1B). The amino acid sequences of OPA1 isoform 1 and isoform 7, henceforth referred to as OPA1 iso 1 and OPA1 iso 7, respectively, were unaltered except for the addition of a 6x HIS tag immediately 5′ of the stop codon. The cDNAs were codon optimized for human codon usage bias in order to maximize potential levels of expression.

### Ectopic Expression of OPA1 iso 1 and OPA1 iso 7 Can Restore Mitochondrial Function

To demonstrate that the optimization process does not perturb mitochondrial localization of the OPA1 iso 1 or OPA1 iso 7 proteins, HEK 293 cells were transiently transfected separately with the two isoforms. When co-stained with an anti-6xHIS tag antibody and Mitotracker orange there was a clear overlap of the two signals, indicating the OPA1 isoforms were successfully targeted to the mitochondria (data not shown).

**FIGURE 2:**
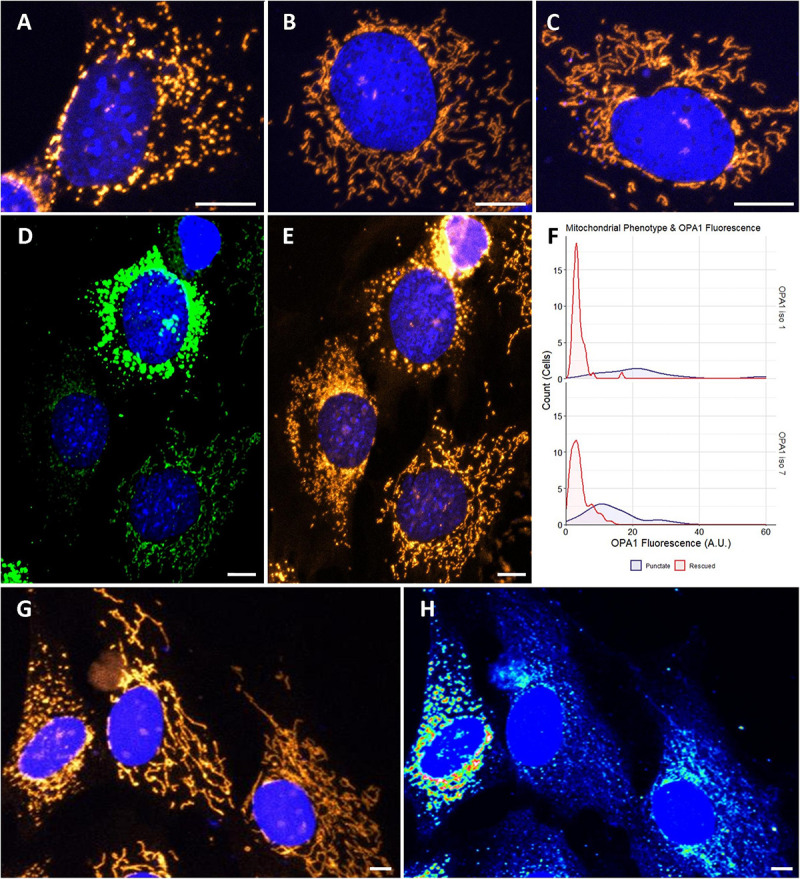
Cytological analysis of OPA1 iso 1 and 7 transient expression. **(A–C)** Show the mitochondrial network of representative OPA1^–/–^ cells, or transiently transfected OPA1^–/–^ with either OPA1 iso 1 or 7 plasmid, respectively. **(D)** A representative image of cells showing widely varying levels of HIS-tagged OPA1 expression. **(E)** Shows that these cells also exhibit distinct mitochondrial phenotypes. **(F)** Cells were grouped by mitochondrial phenotype, demonstrating either punctate (purple) or rescued (red) mitochondria, and the OPA1 fluorescence levels were measured. Only cells that exhibited measurable OPA1 fluorescence were included for this analysis. The two mitochondrial phenotypes demonstrate significantly distinct distributions of OPA1 fluorescence (*n* > 50 cells for each group from 15 FOV over 3 separate transfections, *p* < 0.0001, Wilcoxon rank sum test). **(G,H)** Representative wild type NIH-3T3 MEF cells transfected with OPA1 iso 7. Mitotracker staining in **(G)** demonstrates the gross morphology differences observed with **(H)** showing a heat map of HIS-tagged OPA1 iso 7 protein signal in these cells. This morphological change in the mitochondrial network is seen with OPA1 iso 1 transfection also. Red scale bar = 10 μm.

To investigate if optimized OPA1 iso 1 and OPA1 iso 7 can independently restore mitochondrial morphology, OPA1 knockout MEF (OPA1^–/–^) cells were utilized. These OPA1^–/–^ cells show a characteristic punctate, fragmented mitochondrial network (Figure 2A). In contrast, representative cells transfected with either OPA1 iso 1 or 7 (Figures 2B,C, respectively) show tubular mitochondrial networks indicating restoration of mitochondrial fusion in these cells.

**FIGURE 3:**
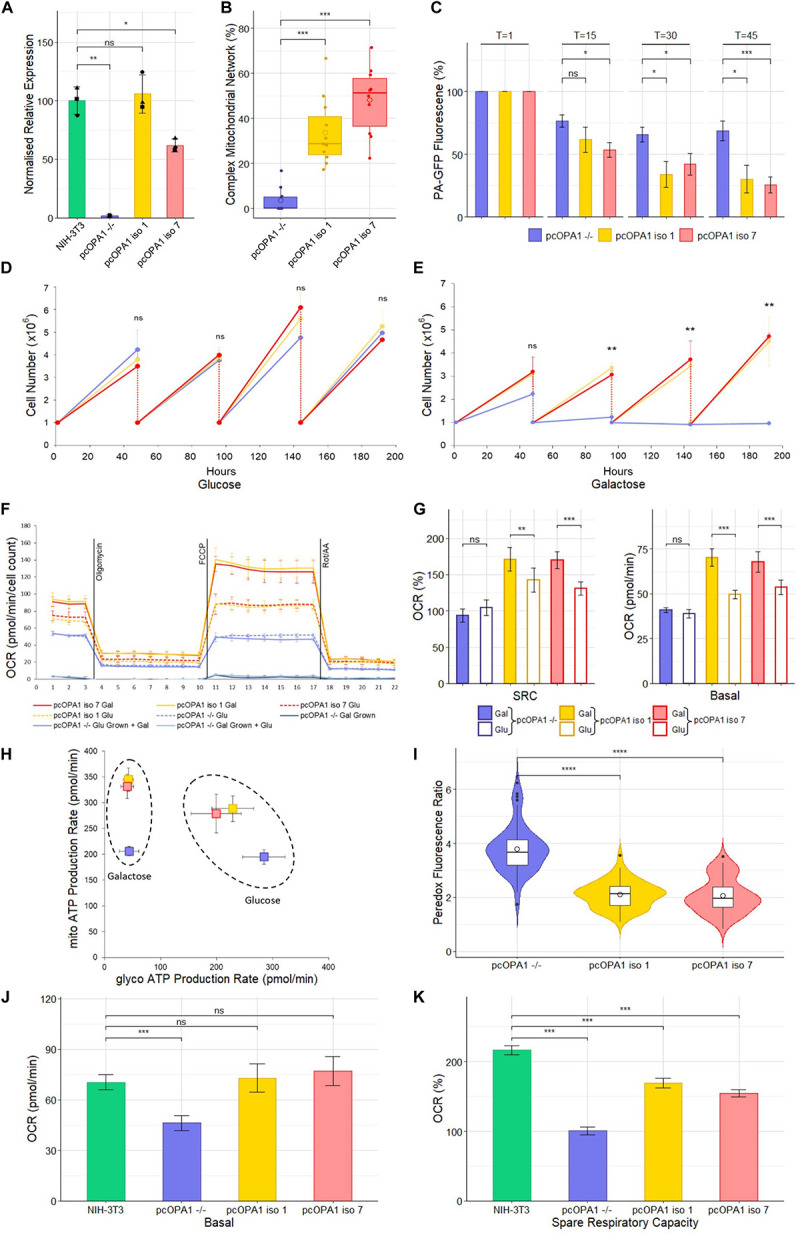
Biochemical analysis of pcOPA1 iso 1 (yellow) and pcOPA1 iso 7 (red) stable cells compared to pcOPA1^–/–^ (purple) stable cells and wild type NIH-3T3 MEFs (green). **(A)** Western blot densitometry analysis of pcOPA1 iso 1, iso 7, and –/– stable cell lines relative to NIH-3T3 cells. Values have been normalized to β-actin. The •, ▲, and ■ symbols represent samples taken 1, 7, and 14 days apart, demonstrating little variation in protein expression levels over time. (*n* = 3 replicates of each cell type and *n* = 4 technical replicates, error bars ± SD). **(B)** Morphological analysis of mitotracker stained mitochondria showing proportion of cells that showed evidence of complex mitochondrial structure (*n* = 10 random FOV across 3 independent trials). **(C)** Mitochondrial Fission Analysis. PA-GFP fluorescence as a percentage of T = 1 measured at 15, 30, and 45 min post illumination (*n* = 10 cells from 5 FOV, error bars ± SE) Rescued cells show a significant improvement in the rate of mitochondrial fusion compared to pcOPA1^–/–^ cells. **(D,E)** show growth curves of each stable cell line with either glucose or galactose as their primary energy source (*n* = 3 independent trials). **(F)** Seahorse XFe96 Mitochondrial Stress Test of the stable cell lines under different growth conditions. Solid lines represent cells grown in 10 mM galactose, hatched lines represent cells grown in 25 mM glucose media for 48 h prior to the experiment (*n* = 8 replicates each group, normalized to cell count, error bars ± SD). **(G)** Shows the basal OCR and SRC as a% of basal OCR from F), (*n* = 8 replicates each group, normalized to cell count, error bars ± SE) Taken together **(F,G)** demonstrate that pcOPA1 iso 1 and 7 cells glucose showed an increase in both basal and SRC when compared to pcOPA1^–/–^ cells grown in glucose. Furthermore, when pcOPA1 iso 1 or 7 cells were grown in galactose they outperform pcOPA1 iso 1 and 7 cells grown in glucose, suggesting improved ability to remodel mitochondria to meet metabolic demands. **(H)** The ATP Rate Assay demonstrates that pcOPA1 iso 1 and 7 cells can utilize their mitochondria more when forced to do so in galactose media, and favor mitochondrial ATP production more even when grown in glucose. (*n* = 8 replicates, error bars ± SD). **(I)** Peredox analysis suggest a significant decrease in cytosolic NADH levels in glucose grown pcOPA1 iso 1 and 7 cells suggesting less reliance on glycolysis for ATP production. (*n* = 60 cells across 3 separate transfections). **(J,K)** Seahorse XFe96 data for pcOPA1 iso 1 and 7 and control compared to wild type NIH-3T3 MEF cells. Both pcOPA1 isoform expressing cells show basal OCR rescue to wild type levels, but do not fully restore SRC. (*n* = 8 replicates per group, normalized to cell count, error bars ± SD). All statistical comparisons are Kruskal-Wallis with *post hoc* pairwise Wilcoxon rank sum test with BH correction, *p < 0.05, **p < 0.01, ***p < 0.001, ****p < 0.0001

During the morphological analysis of the mitochondrial network of these cells it was noted that a number of cells displayed high levels of HIS-tagged OPA1 fluorescence, indicating high levels of OPA1 protein, but also showed a distinctive punctate mitochondrial network morphology, with consistent levels of mitotracker staining (Figures 2D,E, here showing representative OPA1 iso 1 transiently transfected cells). To further examine if this phenomenon was due to OPA1 overexpression 15 fields of view (FOV) were imaged over 3 separate transfections and the morphology of cells were manually scored as being rescued or showing a punctate mitochondrial network distinct from unrescued cells, in cells displaying measurable levels of HIS-tagged OPA1 fluorescence. Figure 3F shows the distribution of the HIS fluorescence varies significantly depending on the mitochondrial phenotype displayed by the cell for both OPA1 iso 1 and OPA1 iso 7 transiently expressing cells (*n* = > 50 cells for each morphology, *p* < 0.001 for both isoforms).

To investigate if this phenotype was due to OPA1^–/–^ cells having a specific sensitivity to OPA1 iso 1 and 7 mediated over-expression, NIH-3T3 MEF cells were transiently transfected with OPA1 iso 1 or 7. Figure 2G shows representative MEF cells labeled with Mitotracker orange, with Figure 2H showing a heat map of HIS-tagged OPA1 iso 7 protein fluorescence demonstrating that mitochondrial morphology is altered with increasing levels of OPA1 iso 7 protein. OPA1 iso 1 expressing cells also demonstrated this overexpression phenotype in NIH-3T3 cells.

### OPA1 Isoform 1 and 7 Expression Leads to Bioenergetic Rescue of OPA1 Cells

To modulate expression levels of OPA1 isoform 1 and 7, cell lines were generated from OPA1^–/–^ MEF cells to stably express the optimized OPA1 isoforms, termed pcOPA1 iso 1 and 7, given the varied mitochondrial morphological phenotype observed in the transiently transfected cells. As a control, a pcOPA1^–/–^ cell line was created using an empty pcDNA3.1 plasmid.

Densitometry of Western blot analysis of the stable cell lines (Figure 3A and Supplementary Figure 1) showed that pcOPA1 iso 1 restored 105.8% (± 16.4%, *p* > 0.05, *n* = 3 replicates) of NIH-3T3 OPA1 protein levels whereas pcOPA1 iso 7 cells restored 61.8% (± 5.5%, *p* < 0.05, *n* = 3) of endogenous NIH-3T3 OPA1 protein levels, showing a substantial increase but still significantly lower than wild-type levels. The overlaid dot plot shows protein extracted from cells sampled at three different time points (•: *T* = 0, ▲: *T* = 7, ■: *T* = 14 days).

The stable pcOPA1 iso 1 and 7 cell lines demonstrated a significant improvement in mitochondrial network morphology. These cells, grown in galactose to force the cells to utilize mitochondrial respiration, showed a significant increase in the number of cells whose mitochondria showed morphological rescue (Figure 3B). To examine if this morphological rescue allowed dynamic remodeling of the mitochondrial networks a mitochondrial fusion assay was performed. This assay monitors the rate of dispersal of PA-GFP signal selectively illuminated in a portion of a cell’s mitochondria; the quicker the rate of dispersal, the higher the rate of fusion in the mitochondria. This assay demonstrated a significant restoration in the ability of pcOPA1 iso 1 and 7 cells to diffuse the PA-GFP signal compared to pcOPA1^–/–^ cells, with an average PA-GFP signal of 30.2% ± 10.9% and 25.7% ± 6.3% remaining in pcOPA1 iso 1 and 7 cells, respectively, at T = 45, compared to 68.6% ± 7.8% in pcOPA1^–/–^ cells (Figure 3C, *n* = 10 ROI from 5 FOV, *p* < 0.05). This indicates enhanced mitochondrial fusion mediated by expression of either OPA1 isoform.

To investigate if the stable expression of the two optimized OPA1 isoforms can provide metabolic benefit compared to pcOPA1^–/–^ cells, a number of biochemical assays were performed. Stably expressing cells were passaged for an extended period of time in either 10 mM galactose media or 25 mM glucose media, the former to force cells to utilize mitochondrial respiration in preference to glycolysis. All cell lines performed equally when grown in glucose media for 192 h (Figure 3D). Conversely pcOPA1 iso 1 and 7 cells survived significantly better when grown in galactose media (Figure 3E), with pcOPA1^–/–^ cells showing a significant decrease in cell number after 96 h (3.37 × 10^6^ ± 2 × 10^5^ pcOPA1 iso 1 and 3.07 × 10^6^ ± 1.7 × 10^5^ pcOPA1 iso 7 vs. 1.23 × 10^6^ ± 3.1 × 10^5^ pcOPA1^–/–^ cells, *n* = 3 replicates, *p* < 0.01), indicating improved ability to utilize mitochondrial respiration in pcOPA1 iso 1 and 7 cells.

In addition, the oxygen consumption rate (OCR) of these cells was measured (Figure 3F). Notably when pcOPA1 iso 1 and 7 cells are compared to pcOPA^–/–^ cells in either glucose or galactose growth conditions, pcOPA1 iso 1 and 7 cells demonstrate a far greater ability to modulate their mitochondrial respiration. Both pcOPA1 iso 1 and 7 cells outperform pcOPA1^–/–^ cells when grown in glucose (basal OCR in glu: pcOPA1^–/–^ 38.9 ± 0.84 pmol/min vs. pcOPA1 iso 1 49.7 ± 0.86 pmol/min and pcOPA1 iso 7 53.62 ± 1.5 pmol/min, normalized to cell count, *n* = 8 replicates, *p* < 0.01, Figure 3E). If each cell line is compared when grown in either galactose (gal) or glucose (glu) media, pcOPA1 iso 1 and 7 cells also showed a marked increase in both basal OCR and Spare Respiratory Capacity (SRC) (Figure 3G; basal OCR: pcOPA1^–/–^ glu 38.9 ± 0.84 pmol/min vs. gal 41 ± 0.57 pmol/min (*p* > 0.05), pcOPA1 iso 1 glu 49.7 ± 0.86 pmol/min vs. gal 70.2 ± 1.7 pmol/min (*p* < 0.001), pcOPA1 iso 7 glu 53.62 ± 1.5 pmol/min vs. gal 67.7 ± 2 pmol/min (*p* < 0.001); SRC (% of basal OCR): pcOPA1^–/–^ glu 93.6% ± 3.8% vs. gal 104% ± 3.7% (*p* > 0.05), pcOPA1 iso 1 glu 142.4% ± 6.2% vs. gal 170.6% ± 5.7% (*p* < 0.01), pcOPA1 iso 7 glu 130.7% ± 3.5% vs. gal 169.7% ± 4.1% (*p* < 0.001), normalized to cell count, *n* = 8 replicates). This suggests a much improved ability of pcOPA1 iso 1 and 7 cells to alter their metabolism to meet environmental constraints compared to pcOPA^–/–^ cells. Furthermore, when pcOPA1^–/–^ cells were grown in galactose prior to the Seahorse analysis they demonstrated extremely low OCRs (basal OCR: galactose grown pcOPA1^–/–^ 3.8 ± 0.65 pmol/min) which could not be rescued by providing the cells with glucose during the assay (basal OCR: glucose grown 3.4 ± 0.57 pmol/min).

To examine the reliance of pcOPA1 cells on glycolysis vs. mitochondrial respiration for ATP production, an ATP rate assay was undertaken. When grown in galactose pcOPA1 iso 1 and 7 cells showed an increase in ATP production from mitochondrial processes compared to pcOPA1^–/–^ cells indicating rescued mitochondrial function (Figure 3H, mito-ATP production in galactose: pcOPA1^–/–^206 ± 8.8 pmol/min, pcOPA1 iso 1 345.1 ± 22.5 pmol/min, pcOPA1 iso 7 331.6 ± 23.5 pmol/min). Interestingly this ability to use mitochondria for ATP generation is still favored by pcOPA1 iso 1 and 7 cells when grown in glucose media, with an increase in mitochondrial ATP production (mito-ATP production glucose: pcOPA1^–/–^ 195 ± 13 pmol/min, pcOPA1 iso 1 279.1 ± 37.6 pmol/min, pcOPA1 iso 7 288.7 ± 24.7 pmol/min) and a corresponding decrease in glycolytic ATP production (glycol-ATP production glucose: pcOPA1^–/–^ 284.7 ± 37.8 pmol/min, pcOPA1 iso 1 199.7 ± 44.7 pmol/min, pcOPA1 iso 7 229.1 ± 37.4 pmol/min).

To confirm this reduction in reliance of glycolysis in pcOPA1 iso 1 and 7 cells, the Peredox fluorescent protein assay was employed (Hung et al., 2011). Peredox protein increases its fluorescence in response to cytosolic NADH levels, with higher levels of cytosolic NADH being indicative of increased levels of glycolysis. pcOPA1 iso 1 and 7 cells grown in glucose show significantly lower levels of Peredox NADH mediated fluorescence (pcOPA1^–/–^ 3.8 ± 0.9 vs. pcOPA1 iso 1 2.1 ± 0.5 and pcOPA1 iso 7 2.1 ± 0.6 normalized fluorescence ratio, *n* = 55 cells observations across 3 separate transfections, *p* < 0.0001), suggesting these cells have a decreased reliance on glycolysis for ATP production compared to pcOPA1^–/–^ cells (Figure 3I).

The mitochondrial activity of pcOPA1 iso 1 and 7 was compared to wild-type (wt) MEF levels grown in galactose media. Notably, rescued cells had no statistical difference in basal OCR when normalized to cell count (Figure 3J; Basal OCR: NIH-3T3 70.3 ± 4.4 pmol/min, pcOPA1 iso 1 72.8 ± 8.3 pmol/min, pcOPA1 iso 7 77.1 ± 8.5 pmol/min, normalized to cell count, *n* = 8 replicates, *p* > 0.05) although all three showed a significant improvement compared to pcOPA1^–/–^ cells (Basal OCR: pcOPA1^–/–^ 46.2 ± 4.6 pmol/min, *n* = 8 replicates, *p* < 0.001). However, there was a significant difference in the spare respiratory capacity (SRC) of rescued cells compared to wt MEF cells (Figure 3K; SRC as% of baseline: NIH-3T3 216.4% ± 6.5%, pcOPA1 iso 1 168.9% ± 7.1%, pcOPA1 iso 7 154.5% ± 5%, pcOPA1^–/–^ 100.8% ± 5.4%).

### AAV delivered OPA1 iso 1 and OPA1 iso 7 Show Benefit in a Mouse Model of Retinal Mitochondrial Dysfunction

Previously our lab has utilized an established rotenone model of complex I deficiency which phenotypically resembles optic neuropathies such as DOA and Leber Hereditary Optic Neuropathy due to significant RGC loss leading to RNFL thinning and a progressive decrease in visual acuity (Zhang et al., 2002; Chadderton et al., 2013).

**FIGURE 4:**
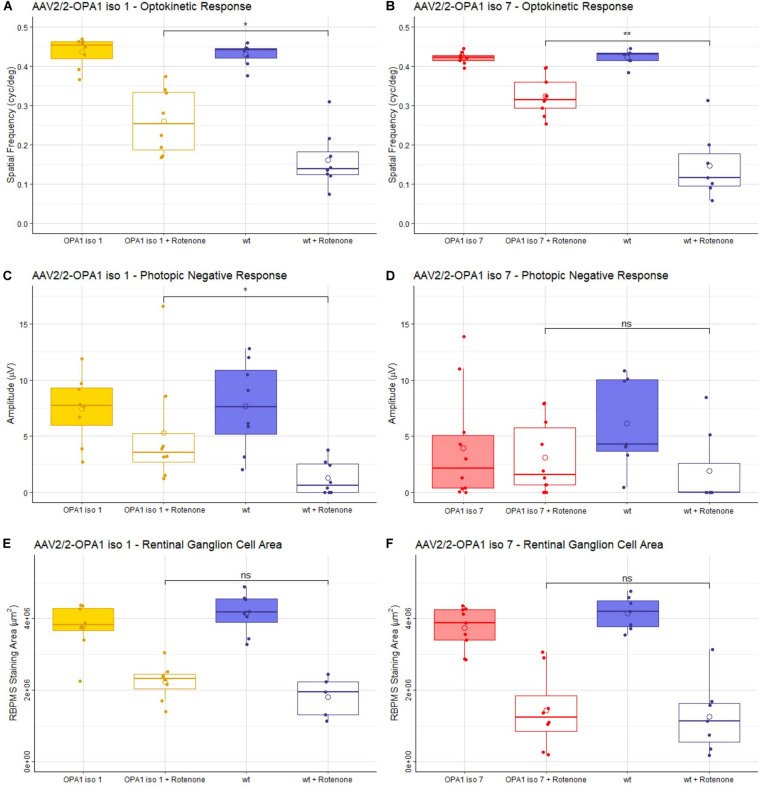
Analysis of AAV2/2-OPA1 iso 1 and AAV2/2-OPA1 iso 7 treatment in a rotenone induced model of optic neuropathy. Adult 129 S2/SvHsd mice were intravitreally injected in both eyes with 1 × 10^9^ vg of either AAV-OPA1 iso 1 or AAV-OPA1 iso 7 and 1 × 10^8^ vg of AAV-CBA-EGFP, 3 weeks post AAV delivery 0.6 μl 1.5 mM rotenone was administered intravitreally. **(A,B)** optokinetic response, maximum spatial frequency (cyc/deg), measured 3 weeks post-rotenone administration. Rotenone treated wt animals in both cohorts show a significant decrease in OKR which is significantly improved by AAV2/2-OPA1 iso 1 or 7 treatment (*p* < 0.05 and *p* < 0.01, respectively). **(C,D)** Show the photopic negative response (PhNR) measured 2 weeks post-rotenone. AAV2/2-OPA1 iso 1 treated animals administered with rotenone show significant protection of PhNR amplitudes compared to wt animals given rotenone (*p* < 0.05), however, AAV2/2-OPA1 iso 7 treated eyes administered with rotenone only showed a modest trend toward protection (*p*≈ 0.2). **(E,F)** Show the area of RBPMS staining (μm^2^) as a measure of RGC cell survival. Neither treatment showed statistically significant benefit in RGC area, however, AAV2/2-OPA1 iso 1 should a modest trend toward protection (*p* ≈ 0.2). Open circle = mean, OPA1 iso 1 and 7 ± rotenone; *n* = 7; wt ± rotenone, *n* = 8 (OPA1 iso 1 cohort) and wt ± rotenone, *n* = 10 (OPA1 iso 7 cohort) pairwise Wilcoxon rank sum test with BH correction (*p < 0.05, **p < 0.01).

To investigate the utility of OPA1 iso 1 and OPA1 iso 7 as therapeutic entities, recombinant AAV vectors were produced and evaluated in the rotenone induced mouse model of mitochondrial dysfunction. The AAV2/2 serotype was chosen for the study due to its ability to transduce RGCs efficiently (Dudus et al., 1999; Chadderton et al., 2013). Adult 129 S2/SvHsd mice were intravitreally injected with 1 × 10^9^ vg of AAV2/2-OPA1 iso 1 or AAV2/2-OPA1 iso 7 followed by injection of the complex I inhibitor rotenone 3 weeks later. After 2 weeks mice were analyzed for visual function (Figure 4). As AAV2/2-OPA1 iso 1 and AAV2/2-OPA1 iso 7 were injected at different times, each is considered with its respective cohort of controls independently.

The optokinetic response (OKR) is a measure of the visual tracking response elicited by a moving pattern. This pattern can be altered to test the maximum spatial frequency the mouse can observe, measured in cycles/degree. The higher the spatial frequency the better the visual acuity. Critically, mice treated with either AAV2/2-OPA1 iso 1 or AAV2/2-OPA1 iso 7 showed no decrease in OKR when compared to uninjected age matched 129 S2/SvHsd mice [hereon referred to as wild-type (wt)] with a mean of 0.43 ± 0.03 cyc/deg for wt compared to 0.43 ± 0.04 cyc/deg AAV2/2-OPA1 iso 1 treated animals (Figure 4A, *n* = 7, *p* > 0.05) and 0.42 ± 0.02 cyc/deg wt compared to 0.42 ± 0.01 cyc/deg AAV2/2-OPA1 iso 7 treated animals (Figure 4B, *n* = 7, *p* > 0.05). Injection of rotenone significantly decreased the OKR of wt mice compared to untreated wt eyes (wt + rotenone OPA1 iso 1 cohort: 0.15 ± 0.09 cyc/deg, *n* = 8, *p* < 0.01; OPA1 iso 7 cohort: 0.16 ± 0.07cyc/deg, *n* = 7, *p* < 0.001). There was significant protection of spatial visual function when eyes were treated with either AAV vector prior to rotenone insult with AAV2/2-OPA1 iso 1 showing an OKR of 0.26 ± 0.08 cyc/deg (*n* = 7, *p* < 0.05 compared to wt + rotenone) and AAV2/2-OPA1 iso 7 showing an OKR of 0.32 ± 0.05 cyc/deg (*n* = 7, *p* < 0.01 compared to wt + rotenone). AAV treatment with AAV2/2-OPA1 iso 1 or AAV2/2-OPA1 iso 7 demonstrated maintenance of 60.5 ± 19% and 76.9 ± 11.9% of the OKR, respectively, compared to their wt controls, with rotenone treated eyes demonstrating 37.6± 17.7% and 34.9± 20.4% of the OKR in the OPA1 iso 1 and 7 groups, respectively. This represents a large proportion of protection, although the OKR was still significantly lower than wt controls.

The photopic negative response (PhNR) of treated animals was interrogated. PhNRs are an electroretinogram (ERG) measurement obtained under dark-adapted conditions as a measure of inner retinal electrical activity and are used as a selective measure of RGC activity (Figures 4C,D). AAV2/2-OPA1 iso 1 (7.4 ± 3 μV) and AAV2/2-OPA1 iso 7 (3.9 ± 4.9 μV) treated animals show no statistical decrease in PhNR compared to untreated wt eyes (iso 1 cohort: 7.7 ± 4 μV, iso 7 cohort: 6.1 ± 4.1 μV), however there is a clear trend toward decreased PhNR in AAV2/2-OPA1 iso 7 treated eyes. Both groups showed a significant decrease in PhNR amplitudes in eyes injected with rotenone when compared to untreated wt eyes (wt + rotenone OPA1 iso 1 cohort: 1.28 ± 1.5 μV, *n* = 8, *p* < 0.01; OPA1 iso 7 cohort: 1.9 ± 3.5 μV, *n* = 10, *p* < 0.05). Notably, AAV2/2-OPA1 iso 1 treated eyes show a significant benefit in PhNR when insulted with rotenone compared to wt eyes (AAV2/2-OPA1 iso 1 + rotenone: 5.3 ± 5.1 μV, *n* = 7, *p* < 0.05) In comparison AAV2/2-OPA1 iso 7 treated eyes showed a modest trend toward benefit (AAV2/2-OPA1 iso 7 + rotenone: 3.1 ± 3.2 μV, *n* = 7, *p* ≈0.2) but displayed more variability within the treated group. As AAV2/2-OPA1 iso 1 treated eyes (without rotenone insult) maintained 96% of the PhNR amplitude of wt animals and AAV2/2-OPA1 iso 7 treated only maintained 64% of wt PhNR it suggests there may have been a modest detrimental effect of AAV2/2-OPA1 iso 7 treatment at this dose.

Whole mounts of the experimental retinae stained for RBPMS, a marker for RGCs, were analyzed to assess if AAV2/2-OPA1 iso 1 and AAV2/2-OPA1 iso 7 retinae showed increased protection of RGCs, by measuring the total area of RBPMS positive staining (Figures 4E,F and Supplementary Figure 2 shows representative wholemount images). Treatment with either AAV-OPA1 construct did not lead to significant changes in RGC area when compared to untreated wt retinae (AAV2/2-OPA1 iso 1: 3.75 ± 0.69 mm^2^ (*n* = 7) vs. wt 4.14 ± 0.55 mm^2^ (*n* = 8), *p* > 0.05; AAV2/2-OPA1 iso 7: 3.73 ± 0.59 mm^2^ (*n* = 7) vs. wt 4.15 ± 0.46 mm^2^ (*n* = 10), *p* > 0.05). Treatment of wt eyes with rotenone led to significant loss of RGCs (wt + rotenone OPA1 iso 1 cohort: 1.81 ± 0.57 mm^2^, *n* = 10, *p* < 0.01; wt + rotenone OPA1 iso 7 cohort: 1.26 ± 1 mm^2^, *n* = 8, *p* < 0.001) which neither AAV2/2-OPA1 iso 1 (2.23 ± 0.5 mm^2^, *n* = 7, *p* ≈0.2) nor AAV2/2-OPA1 iso 7 (1.43 ± 1 mm^2^, *n* = 7, *p* > 0.05) treatment was able to protect from, however AAV2/2-OPA1 showed a modest trend toward RGC protection.

### AAV Treatment Shows Benefit in Primary DOA Patient Derived Fibroblasts

**FIGURE 5:**
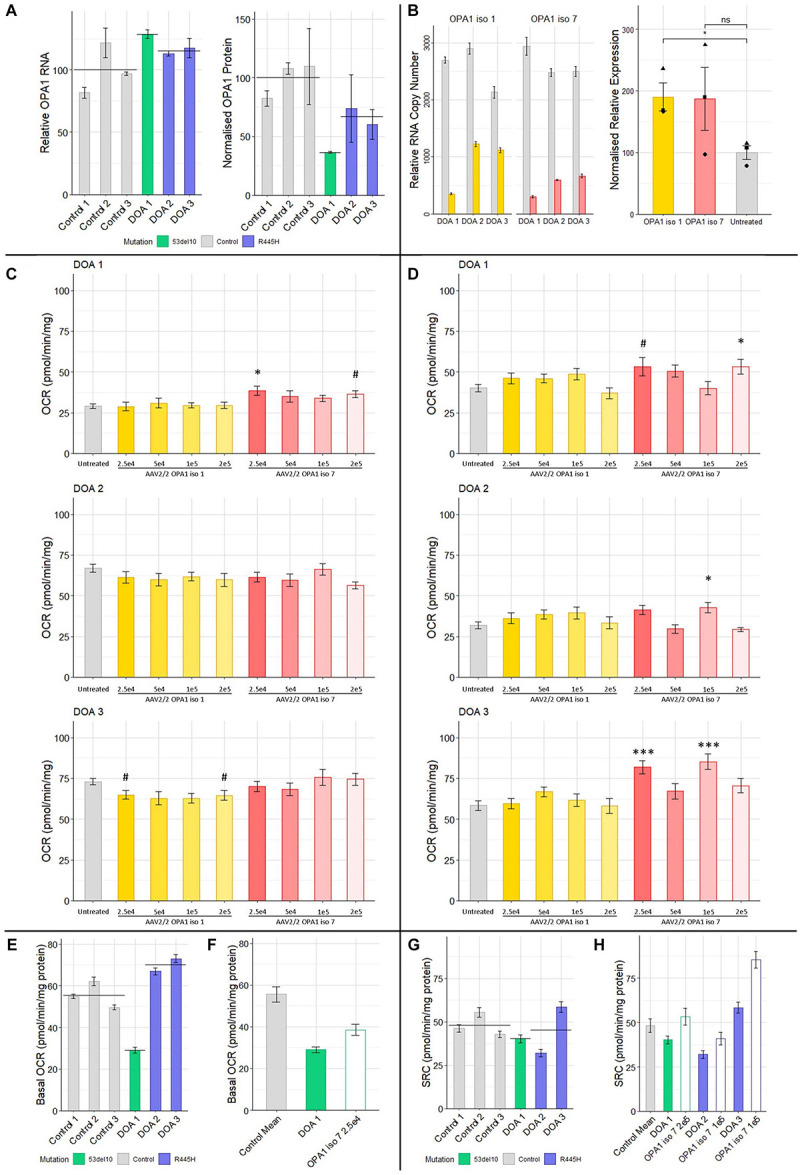
**(A)** Protein and RNA analysis of 3 DOA fibroblast lines (het 53del10: green and het R445H: purple) and 3 control fibroblast lines (gray). RNA (*n* = 4 technical replicates) and protein (*n* = 4 technical replicates) levels are relative to the mean of control samples. **(B)** RNA and protein analysis from patient cells treated with 1 × 10^5^ MOI of AAV-OPA1 iso 1 or 7. RNA samples are relative copy numbers for each construct (*n* = 4 technical replicates) and compare endogenous RNA from all OPA1 isoforms (gray) and transduced OPA1 iso 1 (yellow) or 7 (red) RNA. Densitometry analysis of transduced cells is normalized to the untreated mean for all 3 patient cell lines and shows the mean of all three cell lines for a given treatment (*n* = 3 replicates per cell line, •, ▲, and ■ represent DOA 1, 2, and 3, respectively). **(C)** Basal OCR and **(D)** SRC analysis of three DOA patient derived fibroblast lines, DOA 1 (het 53del10) DOA 2 (het R445H) and DOA 3 (het R445H), grown in galactose either untreated (gray) or treated with four MOIs of either AAV2/2-OPA1 iso 1 (yellow shades) or AAV2/2-OPA1 iso 7 (red shades) 48 h prior to analysis (Seahorse XFe96). OCR was normalized to protein content. SRC was measured as the difference between basal and maximal OCR after removal of non-mitochondrial OCR. (*n* = 3 replicates, Dunnett’s Test vs. Untreated). **(E)** Comparison of the basal OCR of 3 control fibroblast cell lines with the 3 patient cell lines untreated, color coded by mutation (*n* = 3 replicates). **(F)** Comparison of the mean control basal OCR and the only dose found to significantly alter basal OCR from C), 2.5 × 10^4^ MOI AAV-OPA1 iso 7 in DOA 1 cells. **(G)** SRC of control cell lines compared to DOA cell lines, color coded by mutation (*n* = 3 replicates). **(H)** Comparison of the mean SRC of the 3 control lines with untreated DOA SRC values and the viral doses that achieved the largest increase in SRC for each from **(D)** [outlined bars (*n* = 3 replicates)]. ^#^*p* < 0.1, ^∗^*p* < 0.05, ^∗∗∗^*p* < 0.001, error bars ± SE.

To investigate if AAV mediated delivery of OPA1 iso 1 or 7 could improve mitochondrial function in a DOA model, patient derived fibroblasts were generated from 3 DOA patients. DOA 1 (46 y/o, Female) is heterozygous for 53del10 deletion and DOA 2 (49 y/o, Female) and DOA 3 (18 y/o, Female) represent two generations from the same family and are heterozygous for the 1334G > A mutation which leads to an R445H substitution. To act as controls 3 age matched control fibroblast cell lines were used. These donors showed no indication of either mitochondrial or ocular deficits. Figure 5A shows endogenous OPA1 mRNA levels as well as Western blot densitometry analysis of OPA1 protein normalized to β-actin levels in each of the 6 cell lines (*n* = 4 technical replicates, see Supplementary Figure 3 for representative blot). Although mRNA levels remain similar between DOA patients and controls, all three patient cell lines appear to show a deficit of OPA1 protein compared to the control lines, with DOA 1 (53del10) showing the largest decrease. To test the feasibility of AAV2/2-OPA1 mediated therapy all three patient cell lines were treated with 1 × 10^5^ MOI of either AAV2/2-OPA1 iso 1 or 7 before levels of OPA1 mRNA and protein were assessed (Figure 5B). Densitometry was performed on total OPA1 protein normalized to β-actin protein levels and for statistical analysis all DOA samples treated with a given virus were combined. Figure 5B shows there is a significant increase of total OPA1 protein in cells treated with OPA1 iso 1 virus, but this did not quite reach significance with OPA1 iso 7 treated cells.

These fibroblasts were treated with multiple MOI doses of AAV2/2-OPA1 iso 1 and 7 ranging from 5 × 10^4^ to 2 × 10^5^ in 5-fold increments and were subjected to Seahorse analysis of OCR as a dosing study to test if any functional benefit could be observed. Figures 5C,D shows the basal OCR and Spare Respiratory Capacity of these cells normalized to protein content. The basal OCR of these cells remains largely unaltered by AAV treatment apart from DOA 1 cells treated with AAV2/2-OPA1 iso 7 at an MOI of 2.5 × 10^4^ (Figure 5C, 38.5 ± 2.6 pmol/min/mg protein, Dunnett’s test, *n* = 3 replicates, *p* < 0.05).

When examining the SRC of treated DOA patient cells (Figure 5D, only AAV2/2-OPA1 iso 7 treated cells showed significant benefit when compared to untreated, with AAV2/2-OPA1 iso 1 having no significant positive or negative affect in our study. DOA 1 showed maximal benefit to SRC when treated with the highest dose of 2 × 10^5^ MOI (53.3 ± 4.6 pmol/min/mg protein, Dunnett’s test, *n* = 3 replicates, *p* < 0.05). DOA 2 showed significant improvement of their SRC at 1 × 10^5^ MOI (40.8 ± 3.4 pmol/min/mg protein, Dunnett’s test, *n* = 3 replicates, *p* < 0.05). Of note, DOA 3 showed benefit at two separate MOIs, 2.5 × 10^4^ and 1 × 10^5^ (81.8 ± 3.9 pmol/min/mg protein and 85.3 ± 4.6 pmol/mg/protein respectively, Dunnett’s test, *n* = 3 replicates, *p* < 0.001) but not with the MOI in between of 5 × 10^4^.

Figure 5E shows the basal OCR of the 6 different fibroblast lines, color coded by genotype (gray: controls, green: 53del10, purple: R445H, and black bar indicates the group mean). There is a large deficit in basal OCR in DOA 1 cells harboring 53del10. Intriguingly there appears to be an increase in the basal OCR of the R445H cell lines, DOA 2 and 3 which show 120.5 and 131.5% of control cell basal oxygen consumption respectively. To further demonstrate the beneficial effect observed with AAV2/2-OPA1 iso 7 treatment, Figure 5F shows a comparison between the average basal OCR of the three control lines compared to that of DOA 1 cells, as well as the DOA 1 cells treated with 2.5 × 10^4^ AAV2/2-OPA1 iso 7. Before treatment DOA 1 showed 52.2% of the basal OCR of the control cells, with AAV treatment increasing this to 69.4%.

Figure 5G shows the SRC of control cells compared to the DOA cell lines. Both DOA 1 and DOA 2 show a reduction in SRC when compared to control cell lines, however DOA 3 shows high levels of SRC. When comparing the level of increase in SRC AAV-OPA1 treatment mediates (Figure 5H), DOA 1 increases from 83.3 to 120.6% of control levels, DOA 2 increases from 66.3 to 84.7% of control levels and DOA 3 increases from 121.2 to 177% of control levels.

## Discussion

The current study provides clear support for the value of OPA1 based gene therapies for DOA and potentially more broadly for other ocular disorders involving mitochondrial dysfunction. These results suggest that OPA1 isoforms 1 and 7 are the prevalent isoforms expressed in healthy retinal tissue and that both isoforms can be used as potential therapeutic modalities, but consideration needs to be given to ensure appropriate dosing of OPA1 protein. Notably, codon optimized versions of OPA1 isoform 1 and 7 were generated and both isoforms were shown to modulate mitochondrial bioenergetics in cell models of OPA1 dysfunction and protect spatial visual function and PhNR responses in a chemically induced mouse model displaying many of the morphological and functional phenotypes of optic neuropathy, although the OPA1 based therapy only provided a trend toward protection of RGCs. Of note, we demonstrated for the first time that AAV-OPA1 therapeutic interventions can alter mitochondrial bioenergetics in DOA patient derived fibroblast cells, with both OPA1 isoforms evaluated demonstrating the ability to significantly alter mitochondrial bioenergetics by increasing mitochondrial SRC. Taken together, these data underscore the potential utility of OPA1 based gene therapy.

Initial analysis of the OPA1 isoforms expressed in healthy human retinal tissue (Li et al., 2014), representing the first time human retinal tissue has been directly assessed, corroborated with previous studies showing isoforms 1 and 7 to be most prevalently expressed in human tissue in general. It is worth noting that isoform 5 was previously identified as the most highly expressed in human brain tissue (Olichon et al., 2007). In the data analyzed here this was not the case but intriguingly there was a difference in isoform 5 expression levels when comparing the peripheral and macular retinal samples. This difference could be due to the different cellular composition across the human retina, with differences in rod and cone photoreceptor density and cells such as RGCs becoming less prevalent toward the retinal periphery (Drasdo et al., 2007). RNAseq data stratified by cell type would help illuminate the exact isoform distribution in RGCs, however previous analysis of OPA1 isoforms in rat RGCs suggested there was no significant difference in isoform expression patterns between RGCs and the retina at large (Kamei et al., 2005).

The correlation observed between RNA transcript levels involved in mitochondrial fission and fusion suggests that relative expression levels of mitochondrial remodelers may be important to balance mitochondrial network morphology. It is plausible that the increased OPA1 expression provided by transient expression of either OPA1 isoform tested here led to an aggregated mitochondrial network frequently observed due to shifting the balance of fission and fusion. This phenotype has previously been suggested to be due to a compensatory increase in mitochondrial fission (Misaka et al., 2002; Cipolat et al., 2004). Our study highlights the necessity to regulate expression levels of OPA1 tightly in order to provide the maximal benefit to the recipient cell.

Once appropriate levels of OPA1 protein can be achieved there is clear potential for benefit to mitochondrial outputs, as demonstrated by analysis of OPA1 isoform 1 and 7 OPA1^–/–^ MEF stable cell lines. Codon optimization of OPA1 isoform 1 and 7 did not perturb their function as OPA1^–/–^ MEF cells stably expressing either isoform could use mitochondria for ATP production significantly better than OPA1^–/–^ cells, and moreover, OCR under normal physiological conditions could be restored to wild type levels. In terms of their bioenergetic profiles, the two isoforms provide similar levels of functional rescue using these assays. However, pcOPA1 iso 7 cells do show an increased rescue of mitochondrial network morphology (Figure 3A).

Importantly, this study represents the first demonstration that AAV-OPA1 based gene therapy can maintain visual function, evaluated here by OKR. However, just a trend to a corresponding protection of RGCs was obtained. There appears to be a disparity between the two isoforms tested in ability to protect against rotenone insult, with OPA1 iso 1 also demonstrating protection of the PhNR response in treated animals—although a trend toward PhNR protection can also be seen in OPA1 isoform 7 treated animals. One could hypothesize that the disparity between OKR protection compared to PhNR may be due to preferential protection of subsets of RGCs involved in movement, allowing visual function to be preserved whilst there is still a significant reduction in overall RGC activity. It is also noteworthy that AAV-OPA1 iso 7 treatment appears to have had a more dramatic effect on the PhNR response in these animals in the absence of rotenone when compared to AAV-OPA1 iso 1 treatment alone. This is also interesting given that there was no reduction in RGC area between OPA1 iso 7 treated and untreated animals, suggesting cell survival and functionality may be separable. In this study, at equal doses, the two isoforms do not provide equal levels of protection.

Previous AAV-OPA1 based therapeutic studies have shown some protection of RGCs (Sun et al., 2016b; Hu et al., 2018; Sarzi et al., 2018). While this was not recapitulated in our study, there is a trend toward increased RGC number in the rotenone treated AAV-OPA1 isoform 1 cohort compared to wild type mice treated with rotenone. The data suggest that despite levels of RGC loss being similar in all rotenone treated groups, the function of the remaining RGCs is enhanced by both of the OPA1 isoforms, allowing for maintenance of a significant level of visual function. Further work is needed to examine potential dosage levels of each isoform to establish whether both functional and histological benefits can be achieved.

The rotenone model presented here is not directly analogous to a DOA animal model as the insult is acute rather than a prolonged retinal degeneration. Although the model does recapitulate the preferential RGC dysfunction, the mechanism of rotenone induced degeneration is different from that seen in DOA, namely the inhibition of complex I of the electron transport chain vs. OPA1 haploinsufficiency (Alavi et al., 2007; Davies et al., 2007). As such the data here show that OPA1 based gene therapy potentially has a wider applicability as a therapeutic agent for mitochondrial disorders outside of its potential utility in DOA.

The analysis of DOA patient derived fibroblasts underscores the differing effects different viral doses of AAV-OPA1 isoforms can enact and also highlights the range of effects different OPA1 mutations can have on mitochondrial function. Previous analysis of fibroblasts derived from DOA patients with the OPA1 R445H have also found an increase basal OCR over that of control cells, with the hypothesis proposed that basal OCR is increased in these cells as a compensatory mechanism (exact age at time of all biopsies unclear; mean age 23 for 3 patients, *n* = 5; Amati-Bonneau et al., 2005). Furthermore, the FCCP mediated, maximal respiration and spare respiratory capacity of a fibroblast line with the R445H mutation has previously been shown to be significantly increased compared to control fibroblasts, but no increase in basal OCR was observed (age 30; Kane et al., 2017). However this situation is not so clear cut as R445H has also been found to be one of the most deleterious mutations in patient derived fibroblasts, with cells exhibiting lower, but not significantly so, basal and maximal respiration compared to controls (mean age: 38; *n* = 2; Del Dotto et al., 2018b). It is possible that age is a modulating factor for the phenotypic variation observed with the R445H mutation, as mitochondria have long been associated with age related dysfunction (Jarrett et al., 2010; Sun et al., 2016a; Elfawy and Das, 2019). The two patients with the R445H mutations in this study have a mean age of 33.5, with both displaying higher basal OCR than controls. Age could also potentially explain why the SRC of DOA 2 (49 years) is lower compared to DOA 3 (18 years), and indeed why the SRC of DOA 3 is the highest of all cells assayed in this study. Further work is needed to fully elucidate the underlying biochemical effect of the R445H mutation. It is positive that AAV delivered OPA1 isoform 7 significantly increased the SRC in both DOA 2 and DOA 3 cells, which would in theory allow cells to better modulate their metabolism to meet fluctuating energy demands. In this study, efficacy was related to the viral dose delivered. The youngest patient, DOA 3 (∼30 years younger than the others) demonstrated the highest untreated SRC and the largest level of benefit with two doses of virus, potentially suggesting a higher level of plasticity in mitochondrial dynamics.

Currently no other studies have examined the 53del10 deletion, but our data suggests it leads to haploinsufficiency via a decrease in OPA1 protein levels coupled with a decrease in basal OCR and SRC below that of control fibroblasts. Here we have shown that AAV delivered OPA1 isoform 7 can significantly improve both the basal OCR and SRC of these cells, albeit at different doses.

The increase in SRC is promising for potential DOA therapeutics as previous studies have shown patient derived cells to have OCR deficiencies (Amati-Bonneau et al., 2005; Belenguer and Pellegrini, 2013; Vidoni et al., 2013; Kane et al., 2017; Liao et al., 2017; Del Dotto et al., 2018b). Clearly SRC is part of a broader range of mitochondrial roles that are affected by mutations in OPA1, with some debate as to whether mitochondrial dysfunction or the deficiency in mitochondrial network regulation is the main component in DOA related RGC loss (Del Dotto et al., 2018a), but this study highlights the potential utility of AAV delivered OPA1 isoforms to modulate mitochondrial bioenergetics in patient derived cells.

It is unclear why OPA1 isoform 7 outperformed isoform 1 in DOA patient cells and is in contrast to the greater benefit found with OPA1 isoform 1 in the rotenone model shown here, but this could potentially be due to differing physiological roles performed by the two isoforms, although previous work examining the functions of different isoforms has found limited difference between isoforms 1 and 7 (Del Dotto et al., 2017). It seems plausible that with an altered dose that OPA1 isoform 7 could prove as effective as isoform 1 in the animal model and likewise OPA1 isoform 1 could show similar levels of benefit in patient cells.

The current study supports the view that finely regulated dosage appears to be crucial to provide functional benefit when utilizing OPA1 based therapies, and therefore it may be of great benefit to employ the endogenous *OPA1* promoter for such therapies, however, currently the endogenous *OPA1* promoter remains poorly defined. If of a suitable size, incorporating the endogenous promoter into an AAV-OPA1 expression cassette, or employing genome editing strategies, thereby utilizing the endogenous promoter *in situ*, could potentially much improve ability of the construct to provide appropriate OPA1 protein dosage.

Critically, the study provides clear evidence of rescue of spatial visual function *in vivo* of an AAV-OPA1 based gene therapy and represents a step forward in the use of OPA1 isoforms as therapeutic interventions for DOA and potentially for other optic neuropathies more broadly as seen in the rotenone induced mouse model. Intriguingly, this further adds to the evidence that the neuro protective properties of OPA1 may potentially be utilized in a wide array of scenarios. However, the study also underscores that if OPA1 based therapeutics are to be fully efficacious then careful regulation of dosage needs to be achieved.

## Data Availability Statement

The raw data supporting the conclusions of this article will be made available by the authors, without undue reservation, to any qualified researcher.

## Ethics Statement

The animal study was reviewed and approved by the animal research ethics committee, in Trinity College Dublin. Ref. no. 140514. Ethics approval for patient cells obtained from Royal Victoria Eye and Ear Hospital. Ref. no. RF024/17.

## Author Contributions

DM: experimental design, experimentation, figures, writing, editing. NC and SM-W: experimental design, experimentation, writing, editing. AP, CS, and, PK: experimental design, experimentation. JO’B, LC, and DK: experimentation. PH: experimental design. GF: experimental design, writing, editing, funding. All authors contributed to the article and approved the submitted version.

## Conflict of Interest

The authors declare that the research was conducted in the absence of any commercial or financial relationships that could be construed as a potential conflict of interest.

## Footnotes

1 http://www.rstudio.com/
