# Supplementary material for: Optimized OPA1 Isoforms 1 and 7 Provide Therapeutic Benefit in Models of Mitochondrial Dysfunction
Source: Front Neurosci. 2020 Nov 26;14:571479. doi: 10.3389/fnins.2020.571479 (PMC7726421; doi:10.3389/fnins.2020.571479)
Supplement: Supplementary file 1 [file Data_Sheet_1.PDF]

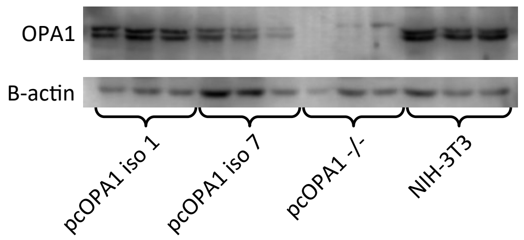

Supplemental Figure 1: Representative Western blot of the three stable cell lines generated compared to NIH-3T3 cells. The three samples for each cell line represent protein samples extracted after 1, 7 and 14 days routine passaging respectively. Densitometric analysis of all 4 technical replicates can been seen in figure 3.

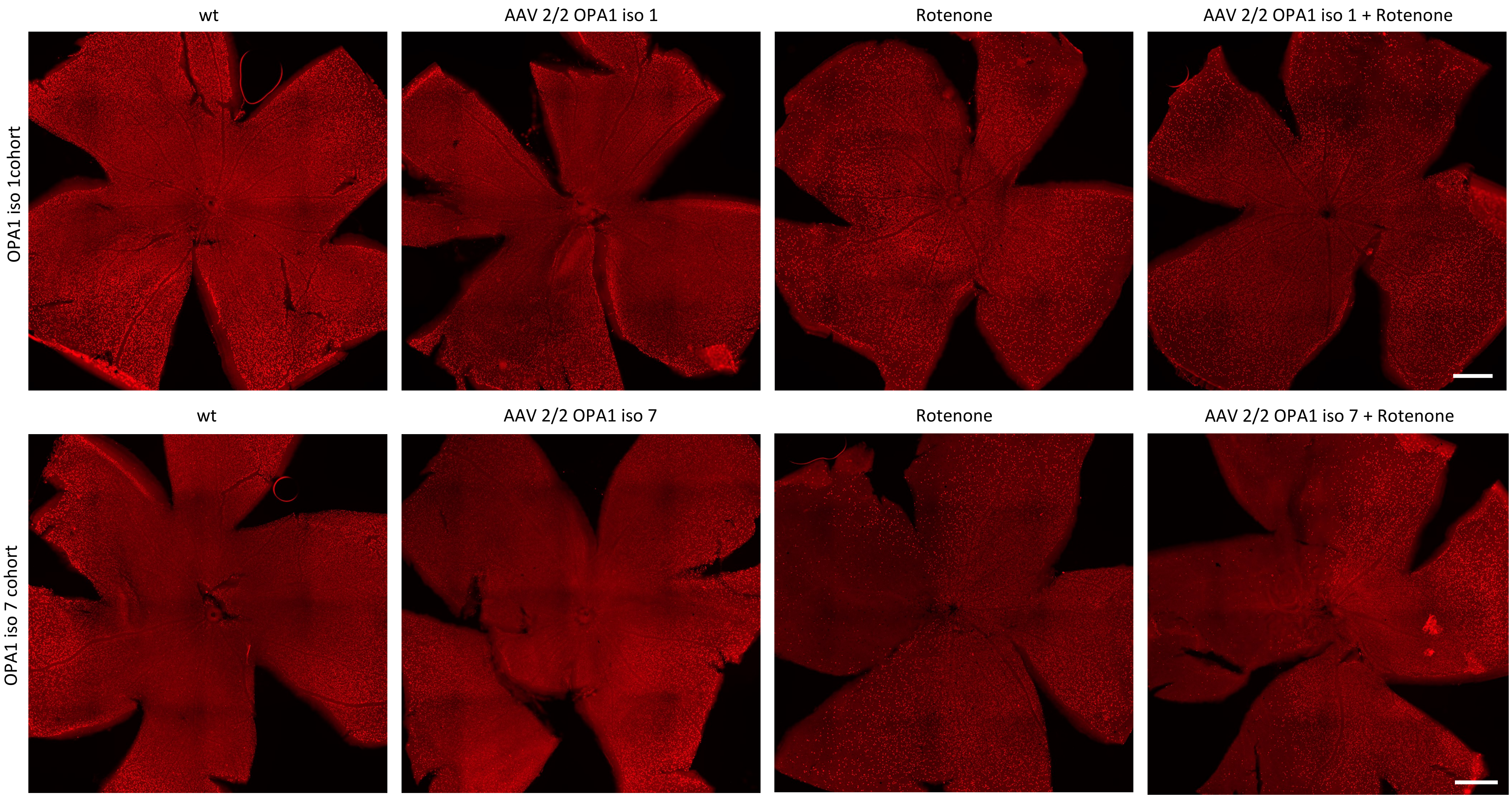

Supplemental Figure: Retinal wholemounts labelled with RBPMS immunocytochemistry to identify RGCs. Two cohorts of mice were treated with either AAV-OPA1 iso 1 or AAV-OPA1 iso 7 bilaterally with rotenone administered to one eye 3 weeks later (n=8). Eyes were fixed and retinal wholemounts labelled with RBPMS primary antibody and then Cy3 conjugated secondary antibody. Lateral microscope images were stitched together to create the wholemount images in cellSens. Representative retinae shown are the closest to the mean of their respective groups. Scale bar = 500 µm.

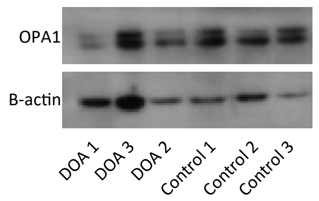

Supplemental Figure 3: Representative Western blot of the 6 fibroblast lines used for the AAV-OPA1 treatment analysis. Densitometric analysis of all 4 technical replicates can be seen in figure 5.

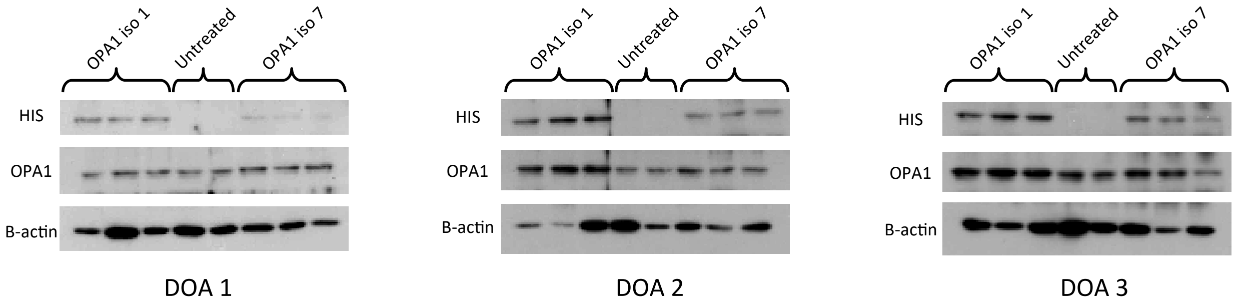

Supplemental Figure 4: Western blots of the three DOA patient cell lines treated with $1x10^5$ MOI of either OPA1 iso 1 or 7 virus. Blots were initially stained with an Anti-6xHIS antibody (top row) before being stripped and re-probed with an OPA1 antibody. Clear expression of HIS-tagged protein can be seen in the treated lanes of each blot but not in the untreated lanes, suggesting successful transduction of the cells. Densitometric analysis of these blots was carried out with the data pooled from all three cell lines for a given treatment (See figure 5).
